# Supplementary material for: The interactome of intact mitochondria by cross-linking mass spectrometry provides evidence for coexisting respiratory supercomplexes
Source: Mol Cell Proteomics. 2017 Dec 8;17(2):216–32. doi: 10.1074/mcp.RA117.000470 (PMC5795388; doi:10.1074/mcp.RA117.000470)
Supplement: Supplemental Data [file supp_RA117.000470_133922_0_supp_23978_fzffwf.zip › spectra_annotation/mito_spectra_annotation/390-1-3-1-7-1.pdf]

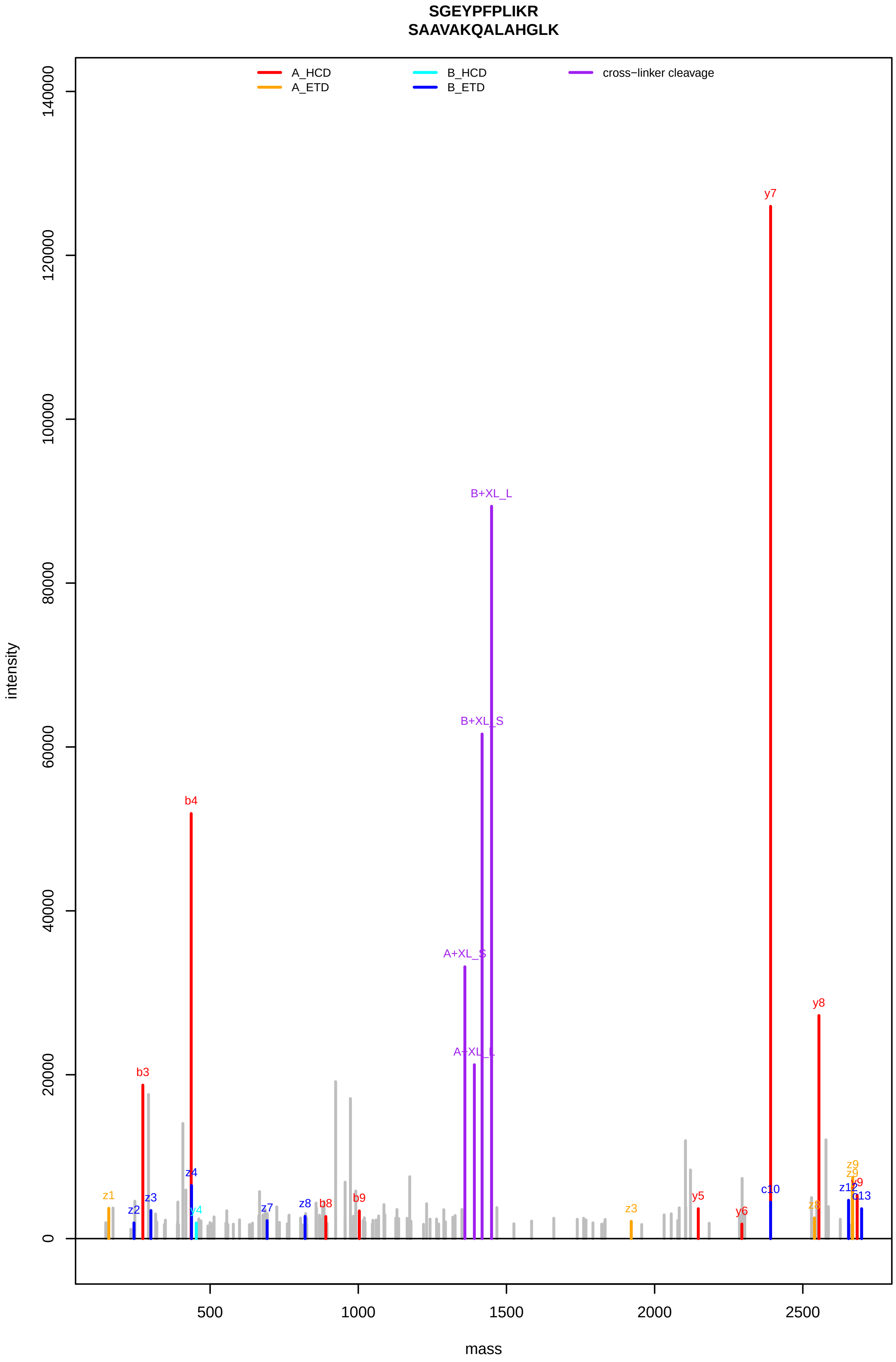
SGEYPFPLIKR
SAAVAKQALAHGLK
A_HCD
A_ETD
B_HCD
B_ETD
cross−linker cleavage
intensity
mass
0
20000
40000
60000
80000
100000
120000
140000
500
1000
1500
2000
2500
y7
B+XL_L
B+XL_S
b4
A+XL_S
A+XL_L
y8
b3
z1
z2
z3
z4
y4
z7
z8
b8
b9
z3
y5
y6
c10
z8
z9
z12
c13

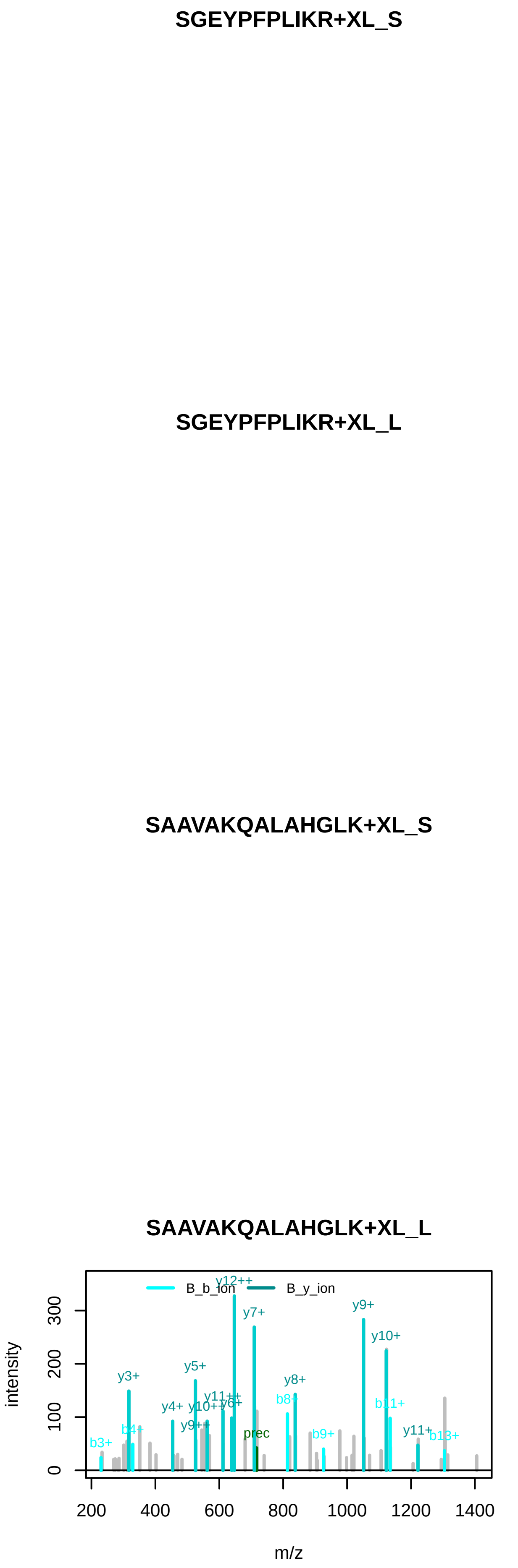
SGEYPFPLIKR+XL_S
SGEYPFPLIKR+XL_L
SAAVAKQALAHGLK+XL_S
SAAVAKQALAHGLK+XL_L
B_b_ion
B_y_ion
intensity
m/z
0
100
200
300
200
400
600
800
1000
1200
1400
y12++
y7+
y9+
y10+
y3+
y5+
y8+
b8+
y4+
y11++
y10++
y6+
y9++
b4+
b11+
b3+
prec
b9+
y11+
b13+
